# What are the factors that affect female healthcare workers on their return to work after maternity leave?

**DOI:** 10.29045/14784726.2025.6.10.1.38

**Published:** 2025-06-01

**Authors:** Jennifer Dod, Gail Lansdown

**Affiliations:** Oxford Brookes University ORCID iD: https://orcid.org/0009-0004-1456-2984; Oxford Brookes University ORCID iD: https://orcid.org/0000-0003-3322-2729

**Keywords:** healthcare professional, maternity leave, return to work

## Abstract

**Introduction:**

The demographic of the paramedic workforce is changing on a worldwide scale, and a higher proportion of paramedics are women of childbearing age. In order to ensure retention in the workplace, it is crucial to understand the challenges faced by female paramedics when returning to work after maternity leave, thus guaranteeing that appropriate support can be provided. Due to a paucity of literature focusing on paramedics, this review has examined literature pertaining to other female healthcare professionals on their return to work and suggests that these factors affect women working in all healthcare disciplines, whether paramedic or otherwise.

**Methods:**

A systematic search strategy using four electronic databases (CINAHL, British Nursing Database, PubMed and Academic Search Complete) was conducted in February 2025. The PRISMA systematic approach was used to conduct a review of the literature, and selected studies were identified using predefined inclusion and exclusion criteria. Papers were narratively synthesised to produce key themes for discussion. As this was secondary research, no ethical approval was required.

**Results:**

A total of 746 records were initially found; after eliminating duplicates and giving consideration to titles and abstracts, 14 remained. Eight of the articles did not meet the inclusion criteria, leaving six, and a further three were found via snowballing and internet searches, giving a final total of nine articles for inclusion in the review. No literature that specifically related to female paramedics was found. Thematic analysis of the papers identified five main themes: work factors, health and well-being, childcare, identity and home support.

**Conclusion:**

The factors that affect female healthcare workers on their return to work after maternity leave are multiple, complex and varied. Women need good support from their workplace in order to have a successful transition back to work, and the availability of childcare for shift workers was problematic. Further research is needed in this area to fully understand the issues facing female paramedics returning to work after maternity leave, as many papers identified were dated, and there is no contemporary UK data pertaining to female paramedics.

## Introduction

The period following the birth of a child is one of huge change and emotional significance for the new mother (Alstveit et al., 2011). Following maternity leave, many women find the transition back to work extremely challenging, with multiple factors influencing a woman’s decision to return to work or not (Alstveit et al, 2011; Coulson et al., 2012; Killien, 2005). During their transition back into the workplace, new mothers need support not only with the practical demands of juggling the care of a young child and work, but also with the emotional and psychological changes that come with the birth of a baby (Ola Naiem & Ruqayya, 2019). For many women, managing childcare and work is difficult, and for those working in healthcare roles such as paramedicine, with long hours and unpredictable finish times, these difficulties are further compounded (Anderson, 2019). Research shows that women in many different professions find a lack of understanding from managers, inflexibility in rotas and lack of general support hamper their return to work after maternity leave (Alstveit at al., 2011; Dean, 2023). Good employer support is a significant factor in influencing a woman’s decision to return to work after maternity leave (Coulson et al., 2012); however, research has shown that women in healthcare roles often struggle with balancing shift work and childcare, and it is often found that workplace support is not adequate (Dean, 2023; Fujimoto et al., 2008; Hill et al., 2023).

The demographic of the ambulance service is changing, and the traditionally male paramedic workforce has seen an increase in female members in recent years (HCPC, 2017, 2021). In 2023, 42% of paramedics registered with the Health and Care Professions Council (HCPC) were women (HCPC, 2023). The increasing popularity of paramedicine as a profession for young women means that the number of female paramedics currently of childbearing age is significant and likely to continue to rise in coming years (Wilson et al., 2022).

Staff retention is a significant issue for the ambulance service (Buchan et al., 2019), and a lack of data about the age at which women leave the paramedic profession and their reasons for doing so has been noted (Wilson et al., 2022). The evolving role of paramedics and increasing availability of jobs outside the ambulance service that involve shorter hours and less shift work means that there are now options for roles that are more family friendly (Eaton et al., 2022).

The aim of this review is to explore and gain a wider understanding of the factors that affect female healthcare workers during their return to work after a period of maternity leave. In order to encourage female paramedics to remain in frontline roles after maternity leave, the ambulance sector needs to address its current policies to acknowledge the issues faced by this staff group. This will enable the sector to evolve effectively as the changing demographic of its workforce provides it with new demands and challenges.

This review initially planned to focus on the factors that affected female paramedics on their return to work from maternity leave; however, the scope was broadened after a lack of literature in that area was revealed. During the course of the review, it became apparent that very little has been written about the difficulties and preferences of women returning to frontline shift work in healthcare roles in the UK. This is surprising considering how much of the NHS workforce is made up of women. This review found only three papers in the past 20 years that explore this topic to any extent for UK healthcare workers, and the most recent UK paper on this topic is over 10 years old. There was no research in this area relating directly to female paramedics.

## Methods

The search terms used (Table 1) were based on the PEO framework (Table 2) developed from the original research question. MeSH terms and a combination of relevant keywords, with appropriate truncation, were used to maximise sensitivity in the search strategy. Boolean operators such as ‘AND’ and ‘OR’ were also employed in the search. Strict exclusion and inclusion criteria (Table 3) were applied to the papers found, allowing the papers to be selected in a uniform, consistent and reliable manner (Garg, 2016). Four chosen databases were searched in February 2025: CINAHL, British Nursing Database, PubMed and Academic Search Complete. All were available via institutional access through the Oxford Brookes University Library website. All the study results obtained via the initial search were screened by reading the title and abstract. After any duplicate studies were identified and removed, the full articles were then assessed against the strict inclusion/exclusion criteria described in Table 3.

**Table 1.** Search terms and synonyms.

Search term 1	Search term 2	Search term 3
Healthcare ‘Healthcare professional’ Nurs* Paramedic* Doctor* Midwi* ‘Health professional*’	Maternity ‘Adoption leave’ ‘Parental leave’ Childbirth	‘Return to work’ RTW ‘Work re-entry’ ‘Resume work’ ‘Returning to work’ ‘Job re-entry’

Words were combined using OR within columns, AND between columns. ‘Phrase searching’ and *truncation were also used.

**Table 2.** The PEO formulation.

Population	Female healthcare workers returning to work after maternity leave.
Exposure	Maternity leave.
Outcome	Factors affecting their return to work after maternity leave.

**Table 3.** Inclusion and exclusion criteria.

**Inclusion**
Papers published after January 2000 – due to lack of recent literature on this topic. Papers written in English – for clarity and understanding. Full-text articles available – so the entire article can be considered. Papers from UK, Australia, New Zealand, Canada and Europe – healthcare provision and maternity leave considerations in these countries are similar to those in the UK, rendering the data transferable. Peer-reviewed papers – to ensure quality.
**Exclusion**
Papers or research from America – the healthcare system and maternity provision in America is so different from that of the UK that data would not be transferable. Studies where participants were not female healthcare workers who have returned or are returning to work after maternity leave – data would not be relevant. Review papers or secondary data – only primary data were used to ensure quality and minimise bias.

In order to ensure all possible literature was found, a snowball search consisting of citation tracking was carried out on the selected papers (Greenhalgh & Peacock, 2005). A further online search was carried out using Google Scholar and Web of Science to ensure that no papers had been missed. The papers were then assessed for quality using the CASP critical appraisal tool (CASP, 2024) for qualitative studies and the MMAT for mixed method studies (Hong et al., 2018). No studies were excluded on the basis of quality.

## Results

**Figure 1:**
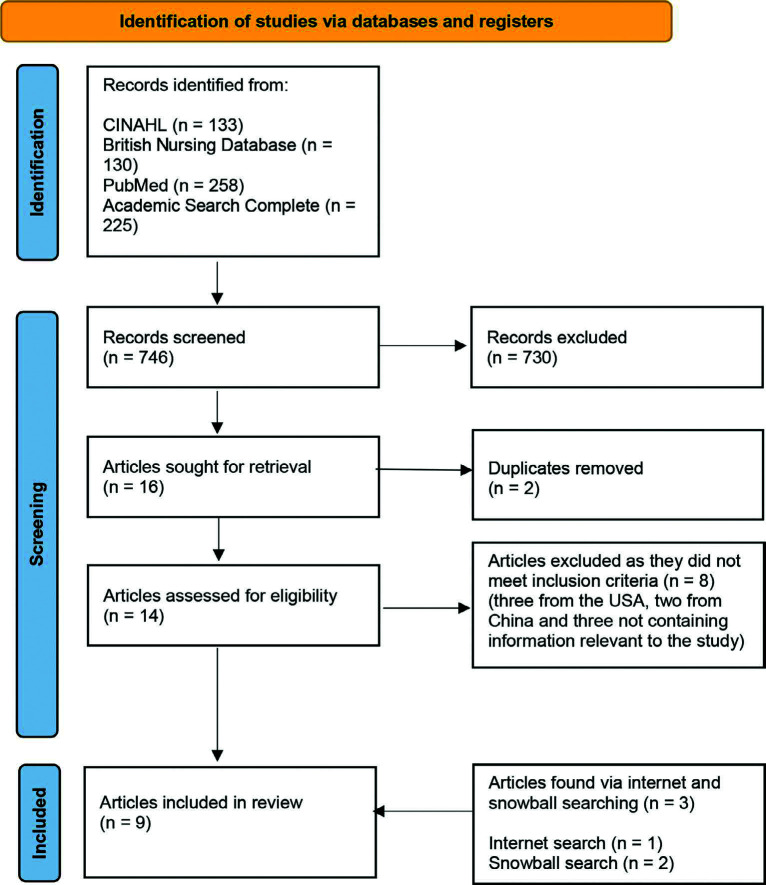
Figure 1. PRISMA flow diagram of the screening process.

Overall, 746 possible papers were found. After title and abstract screening, a total of 16 articles were identified, and after removing duplicates, 14 of these articles remained (Figure 1). One further article was identified from a simple internet search using Web of Science and Google Scholar, and a further two possible studies were identified via snowball searching. Full-text reviews of these 16 papers yielded nine papers for final inclusion in the review. Of the papers not included, three were from the USA and two were from China, therefore not meeting the inclusion criteria, and three did not contain relevant information about the factors affecting women returning to work after maternity leave. Details of the nine included papers can be found in Table 4.

**Table 4.** Included articles.

Author and location	Study aim	Study design and participants	Main findings
**1. Khalil and Davies (2000)** **UK**	To gain understanding of experiences of nurses returning to work after childbirth and to make staff retention recommendations.	Qualitative – semi-structured interviews. 5 nurses	Returning to work was important for career continuation and mental stimulation. Women had to renegotiate their home and work roles after returning to work. Partner and home support were essential.
**2. Walsh et al. (2005)** **Canada**	To determine which factors enable or impede women in a family-medicine residency programme from combining training and motherhood.	Qualitative – semi-structured interviews. 21 family-medicine residents.	Women found long, unpredictable hours and work schedules, attitudes of colleagues and supervisors and finding adequate childcare the main stressors of returning to the workplace after childbirth.
**3. Redwood (2008)** **UK**	To explore nurses’ and midwives’ perceptions of their transition to motherhood and its implications and impact on their jobs.	Qualitative – semi-structured interviews. 22 nurses and midwives.	The transition to motherhood had direct impacts on patient care and work practices. Women encountered difficulties in returning to the workplace, especially around childcare and workplace flexibility.
**4. Nowak et al. (2013)** **Australia**	To explore how responsibilities for childcare were managed on return to work after maternity leave.	Both qualitative and quantitative. Questionnaires were employed with some open-ended questions. 388 healthcare professionals.	Childcare was often provided by family members. Women found difficulties arranging and finding childcare to fit with working hours and shift work. Employers were often inflexible and unsupportive of part-time hours or flexible working.
**5. Brightwell et al. (2013)** **UK**	To explore the experiences of trainee doctors returning to work after maternity leave to identify what support they require.	Qualitative – focus groups. 22 postgraduate doctor trainees.	Women struggled with a number of aspects of returning to work after maternity leave, such as attrition of skills, logistical worries, part-time working and working with sick children.
**6. Parcsi & Curtin (2013)** **Australia**	To gain insight of the experiences of occupational therapists on their return to work after maternity leave.	Qualitative – semi-structured interviews. Six occupational therapists.	Two main themes of compromise and feeling valued were identified. Women made compromises in many different areas on their return to work and all felt that being valued in the workplace was very important.
**7. Landon & Selk (2018)** **Canada**	To identify barriers and support for obstetrics and gynaecology residents taking maternity leave.	Qualitative – semi-structured interviews. 15 obstetrics and gynaecology residents.	Challenges (breastfeeding, support on return, work–life balance) and support (partner, childcare and colleagues) were identified by the women when they returned to work. Gradual returns and careful rota planning were suggested alleviators to the challenges.
**8. Costantini et al. (2022)** **Italy**	To gain an understanding of nurses’ experiences when they returned to work after a prolonged (>12 months) maternity leave.	Qualitative – semi-structured interviews. 12 nurses.	Having a large support network was important for a successful transition back into the workplace. Women found leaving their children difficult but were very motivated to return to work and found they had improved skills in some areas of patient care.
**9. Allen et al. (2024)** **Australia and New Zealand**	To explore the experiences of anaesthetists who had returned to work after maternity leave and identify influential factors.	Qualitative – semi-structured interviews. 15 anaesthetists.	Women found the transition back to work challenging; emotionally, physically and professionally. Workplace support was variable and women often experienced negative attitudes on re-entry.

Of the nine final papers, none included paramedics as participants. Participants were from a range of healthcare backgrounds, including doctors and trainees, nurses, midwives and occupational therapists. The research came from a variety of locations: the UK, Canada, Australia, New Zealand and Italy. Although none of the papers contained data from paramedics, the shift working and high-pressure nature of the jobs of the included participants means that some transferable conclusions can be drawn.

**Figure 2:**
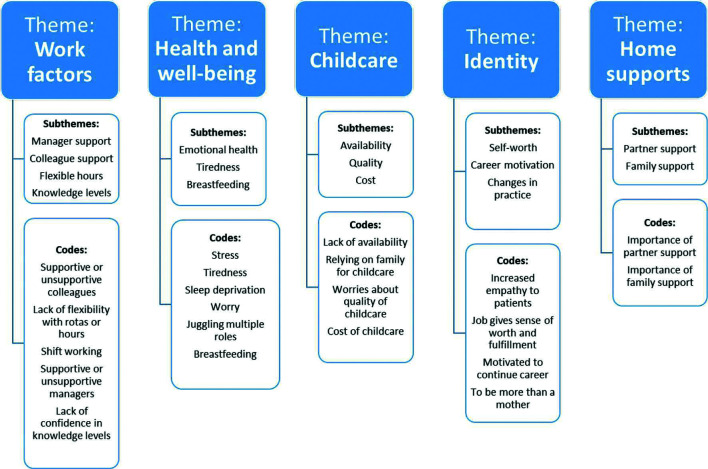
Figure 2. Thematic analysis.

The papers were studied using thematic analysis. After reading and re-reading, codes were organised from the papers into themes, with five main themes identified: work factors, health and well-being, childcare, identity and home support (Figure 2).

## Discussion

### Work factors

Flexible working was mentioned in all the studies. In six of the nine selected studies (Allen et al., 2024; Brightwell et al., 2013; Costantini et al., 2022; Khalil & Davies, 2000; Nowak at el., 2013; Redwood, 2008) the women found difficulties in accessing flexible working, and in three studies (Landon & Selk, 2018; Parcsi & Curtin, 2013; Walsh et al., 2005) the women were positive about their flexible working arrangements. It is worth noting that of those reporting positive flexible working arrangements, one of the studies involved occupational therapists (Parcsi & Curtin, 2013), who do not work 24-hour shifts, and the other two (Landon & Selk, 2018; Walsh et al., 2005) had participants from residency training programmes in Canada, so their generalisability to the UK NHS may be limited. Difficulties in accessing flexible working were experienced by all the women in the studies from the UK, which supports the findings of Robinson et al. (2003) and an NHS Employers (2020) survey, which found that nurses in the UK experienced difficulties in obtaining flexible working patterns on their return to work after maternity leave. The women in the studies who had positive experiences surrounding flexible working stated that accessing flexible working really helped their transition back into work. This is backed up by research from Gould & Fontenla (2006) and McNall et al. (2010), which shows that flexible working arrangements are important to securing work commitment and are associated with greater job satisfaction and fewer turnover intentions. Unsurprisingly, difficulties in accessing flexible working seemed to be heavily linked to manager support. The three papers that cited positive flexible working access also stated a high level of manager support (Landon & Selk, 2018; Parcsi & Curtin, 2013; Walsh et al., 2005). Literature suggests that women returning to work after maternity leave often face significant challenges in accessing part-time roles and flexible working patterns (Dean, 2023; Franzoi, 2024; Hutchings et al., 2023). Manager support is instrumental in facilitating these opportunities and supporting women’s transition back to work. The NHS is currently promoting flexible working arrangements (NHS England, 2022), and research shows that flexible working arrangements are linked to work commitment, engagement and well-being (Gould & Fontenla, 2006).

Lack of manager support was reported in six of the papers (Allen at al., 2024; Brightwell et al., 2013; Costantini et al., 2022; Khalil & Davies, 2000; Nowak et al., 2013; Redwood, 2008), including the three from the UK (Brightwell et al., 2013; Khalil & Davies, 2000; Redwood, 2008). Coulson et al. (2012) found that good employer support impacts women’s decisions on whether to return to work after maternity leave. The NHS is currently struggling with recruitment and retention of healthcare workers (NHS England, 2023), and this review suggests that giving proper managerial support during pregnancy and maternity leave would encourage women to return to their roles and would help maintain staff satisfaction, something that the NHS is currently striving to achieve.

### Health and well-being

Most women found returning to work after maternity leave emotionally challenging, with women stating they suffered stress from juggling multiple roles, trying to achieve a work‒life balance and managing the guilt surrounding returning to work and leaving their child in the care of others (Allen et al., 2024; Brightwell et al., 2013; Costantini et al., 2022; Landon & Selk, 2018; Parcsi & Curtin, 2013; Redwood, 2008; Walsh et al., 2005). This is consistent with the findings of studies by Alstveit et al. (2011), Coulson et al. (2012) and Millward (2006). Tiredness was one of the main issues, with women stating that sleep deprivation and tiredness made their transition back to work more difficult. Studies by Kirby et al. (2016) and Anderson (2019) show that paramedics find both shift working and the nature of their role physically and emotionally tiring and therefore balancing this with the demands of a young child is likely to be very stressful for women returning to the ambulance service after maternity leave.

### Childcare

A general consensus from the studies was that having high-quality childcare and knowing their child was well cared for was very important (Costantini et al., 2022; Khalil & Davies, 2000; Landon & Selk, 2018; Nowak et al., 2013; Parcsi & Curtin, 2013; Redwood, 2008). A recent UK government survey found that 60% of working women said that having reliable childcare helped them go back to work (UK Government, 2022). Five of the included studies mentioned difficulties in finding childcare to fit around work, and all the women who experienced these difficulties were shift workers (Allen et al., 2024; Brightwell et al., 2013; Khalil & Davies, 2000; Nowak et al., 2013; Walsh et al., 2005). A study by Kirby et al. (2016) found that paramedics often rely on family to fulfil their childcare needs and that organising shift working and childcare was problematic. Surprisingly, only two of the studies selected mentioned the cost of childcare, which was deemed expensive or too high (Nowak et al., 2013; Redwood, 2008). The recent cost-of-living crisis and difficulties in accessing pre-school childcare in the UK means that only very recent literature would be likely to accurately reflect current views on the cost of childcare and how this impacts women returning to work after maternity leave.

### Identity

This theme explored the women’s views of themselves, how they felt as mothers and professionals and how this was affected by their maternity leave. Three sub-themes were further identified within this: self-worth, career motivation and changes in practice. Women felt that working boosted their esteem; they looked forward to being themselves at work, felt that work gave them confidence and were happy to contribute towards society (Brightwell et al., 2013; Costantini et al., 2022; Khalil & Davies, 2000; Parcsi & Curtin, 2013). Literature by Alstveit et al. (2011) and Rodrigues et al. (2017) also found that women placed high value on returning to work, especially in the nursing profession, where work is linked to having high self-esteem and is viewed as an important source of social interaction.

In five of the papers analysed (Allen et al., 2024; Brightwell et al., 2013; Costantini et al., 2022; Khalil & Davies, 2000; Landon & Selk, 2018), women were highly motivated to resume their careers and continue on the career pathway that they had worked hard towards. However, in two of the papers (Parcsi & Curtin, 2013; Redwood, 2008), women mentioned that they felt less career motivated than before having children. In their 2005 paper, Davey et al. explored nurses’ motivations for returning to work after maternity leave and found that nurses were highly motivated by work and career. Like nursing, paramedicine is a vocational career, and it is likely that female paramedics will also be highly motivated by their career to return to work after maternity leave and are likely to link work to their sense of identity and self-esteem.

### Home support

Many of the included studies mentioned that good family and partner support were essential for a smoother transition back into the workplace. It was unsurprising that positive support given by partners and family were factors that made women’s transition back to the workplace easier. Evidence (Coulson et al., 2012; Killien, 2005) shows that good family and social support has a positive effect on women’s decisions to return to work after maternity leave and makes their transition back into the workplace easier. With so many families relying on childcare given by grandparents and other family members (UK Government, 2022), it is not surprising that good family support is so important, and in two of the selected studies (Nowak et al., 2013; Redwood, 2008) family support is mentioned as being essential for childcare.

### Limitations

Limitations of this review include the narrative synthesis by a single researcher (JD). This means there is the risk of researcher bias in the discussion points. The lack of UK-based research in this area, especially in relation to paramedics, means that the generalisability of this review to UK paramedic practice is limited. However, the authors feel confident that this review provides a good overview of the current state of research within this field.

## Conclusion

Women in general often find returning to work after maternity leave challenging, and this is no exception for female healthcare workers. This review found that female healthcare workers returning to work faced multiple challenges, and the factors affecting their return to work were numerous and complex. It also highlighted a serious lack of literature in this area, with no primary data on this topic for female paramedics.

This lack of research suggests that ambulance services cannot currently provide adequate support for women returning to frontline roles after maternity leave and are at risk of losing staff to more family-friendly roles. This is especially the case in the UK ambulance sector, where the number of young women qualifying as paramedics is increasing. A lack of focus and understanding of the issues relevant to women returning to work after maternity leave may lead to discrimination against women who have children and has the potential to significantly impact staff retention in the future.

A key finding of this review is that flexible working is essential for women returning to work from maternity leave. Many women found that their workplace either did not offer flexible working or it was difficult to access, with manager support in this area often lacking. Workplaces need to be aware of these challenges and offer appropriate support for those juggling family life with a career in healthcare. Childcare was also an issue for many women. Finding adequate childcare to fit around shift working is difficult, and traditional childcare settings cannot meet the needs of those working unsocial hours. Recent increases in the cost of living, changes in government funding for childcare and fluctuations in childcare provision in different regions mean that up-to-date research is needed to explore how these factors impact women who work shifts. It is clear that the needs and preferences of this demographic are poorly understood, and further research is needed in this area.

This review recommends that primary research is undertaken to explore the experiences and preferences of female paramedics who have returned to work after maternity leave. This should be a detailed exploration of women’s lived experiences, using qualitative methods. A larger and more wide-scale study aimed at obtaining an overview across the profession would also be beneficial. Up-to-date research and information on this topic will allow ambulance service policy to be shaped using current information about staff needs and preferences. Ambulance services worldwide face a difficult task managing the ever-increasing demands on the service and the challenges of running a 24-hour a day, year-round service, as well as meeting the needs of staff with children and families. Currently there is no data to underpin policy making in this area and no understanding of the needs and preferences of women returning to work after maternity leave. Further study in this area will give ambulance services the vital information they need in order to work towards retaining female staff after maternity leave and allowing staff to maintain a better work‒life balance.

## Author contributions

JD completed this review as part of a postgraduate dissertation, with GL as the supervisor. Both reviewed and approved the final manuscript. JD acts as the guarantor for this article.

## Conflict of interest

None declared.

## Ethics

Not required.

## Funding

None.
